# Enhancing Chimeric Antigen Receptor T Cell Anti-tumor Function through Advanced Media Design

**DOI:** 10.1016/j.omtm.2020.07.008

**Published:** 2020-07-09

**Authors:** Saba Ghassemi, Francisco J. Martinez-Becerra, Alyssa M. Master, Sarah A. Richman, David Heo, John Leferovich, Yitao Tu, Juan Carlos García-Cañaveras, Asma Ayari, Yinan Lu, Ai Wang, Joshua D. Rabinowitz, Michael C. Milone, Carl H. June, Roddy S. O’Connor

**Affiliations:** 1 Center for Cellular Immunotherapies, Perelman School of Medicine at the University of Pennsylvania, Philadelphia, PA, USA; 2 Department of Pathology and Laboratory Medicine, Perelman School of Medicine of the University of Pennsylvania, Philadelphia, PA, USA; 3 Nucleus Biologics, LLC, San Diego, CA, USA; 4 Division of Oncology, Department of Pediatrics, Children’s Hospital of Philadelphia and Perelman School of Medicine at the University of Pennsylvania, Philadelphia, PA, USA; 5 Department of Biological Physics, University of Pennsylvania, Philadelphia, PA, USA; 6 Department of Chemistry, Princeton University, Princeton, NJ, USA; 7 Lewis-Singer Institute for Integrative Genomics, Princeton University, Princeton, NJ, USA

## Abstract

Effective chimeric antigen receptor (CAR)-T cell therapy is dependent on optimal cell culture methods conducive to the activation and expansion of T cells *ex vivo*, as well as infection with CAR. Media formulations used in CAR-T cell manufacturing have not been optimized for gene delivery, cell expansion, and overall potency. Bioactive components and derivatives that support the generation of functionally-competent T cell progeny with long-lasting persistence are largely undefined. Current media formulations rely on fetal bovine serum (FBS) or human serum (HS), which suffer from a lack of consistency or supply issues. We recognize that components of blood cellular fractions that are absent in serum may have therapeutic value. Here we investigate whether a concentrated growth factor extract, purified from human transfusion grade whole blood fractions, and marketed as PhysiologixTM xeno-free (XF) hGFC (Phx), supports CAR-T cell expansion and function. We show that Phx supports T cell proliferation in clinical and research-grade media. We also show that Phx treatment enhances lentiviral-mediated gene expression across a wide range of multiplicity of infections (MOIs). We compared the ability of anti-GD-2 CAR-T cells expanded *ex vivo* in medium conditioned with either Phx or HS to clear tumor burden in a human xenograft model of neuroblastoma. We show that T cells expanded in Phx have superior engraftment and potency *in vivo*, as well as CAR-induced cytolytic activity *in vitro*. Metabolomic profiling revealed several factors unique to Phx that may have relevance for CAR-T cell preclinical discovery, process development, and manufacturing. In particular, we show that carnosine, a biogenic amine modestly enriched in Phx relative to HS, enhances lentiviral gene delivery in activated T cells. By limiting extracellular acidification, carnosine enhances the metabolic fitness of T cells, shifting their metabolic profile from an acidic, stressed state toward an oxidative, energetic state. These findings are very informative regarding potential derivatives to include in medium customized for gene delivery and overall potency for T cell adoptive immunotherapies.

## Graphical Abstract

**Figure:**
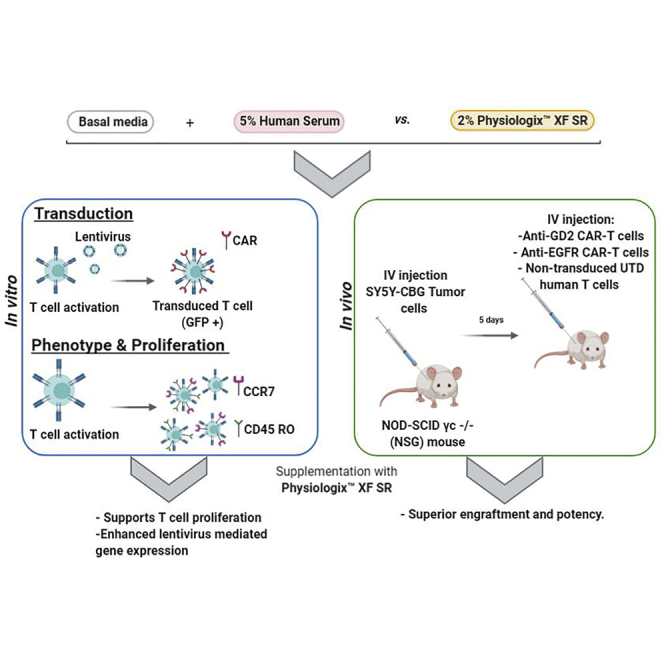

## Teaser

Media formulations used in CAR-T cell therapies have not been optimized for gene delivery, cell expansion, and overall potency. Ghassemi et al. report that anti-GD2 CAR-T cells expanded in medium conditioned with a concentrated growth factor extract have superior engraftment and potency in a human xenograft model of neuroblastoma.

## Introduction

Adoptive immunotherapy provides a promising approach for the treatment of a variety of diseases, including cancer and chronic viral infections. In these therapies, synthetic receptors are often used to redirect T cell specificity against distinct cancer antigens. Modifying T cells with transgenes to bolster their function and expand their corresponding progeny occurs in highly-controlled *ex vivo* settings. In the context of chimeric antigen receptor (CAR) therapies, patient-derived T cells are activated, genetically modified, and expanded over 9–11 days in nutrient-rich conditions.^1^ An overlooked aspect of the *ex vivo* process is understanding how the metabolic composition of the media impacts the functional attributes and behavior of the final cell product.

Activated T cells shift their metabolism from oxidative phosphorylation to aerobic glycolysis to meet increasing biosynthetic demands. The anabolic role of intracellular metabolites and their derivatives in T cell proliferation and differentiation has been well described.^2^ Given that activated T cells synthesize macromolecules including nucleotides, lipids, and proteins *de novo*, they are reliant on an exogenous source of circulating nutrients. As extrinsic factors in the microenvironment impact proliferation, phenotype, and gene transduction, the overall outcome of the *ex vivo* culture process is highly dependent on the properties of the medium.

T cell media are conditioned with serum from either animal or human origin. Serum provides an important source of bioactive peptides, hormones, and growth factors that collectively support cell growth. In designing an optimized medium for T cell therapies, it’s important to understand how serum constituents influence transduction, proliferation, and differentiation. Identifying crucial factors that influence T cell function may lead to the development and inclusion of chemical derivatives as conditioning agents in defined medium formulations. Exemplifying its rich source of trophogens, serum from animal origin such as fetal bovine serum (FBS) is widely used in research applications and preclinical discovery.^3^ However, cell culture with FBS *in vitro* does not mimic human microenvironments. This limits the translational applications of FBS, underscoring the need for effective substitutes. FBS is also unsuitable for cell-based therapies since it carries the risk of transmitting bovine spongiform encephalopathy, as well as viral pathogens. As a conditioning agent for human cells grown *ex vivo* in highly-controlled settings, human serum (HS) has inherent advantages over bovine. HS provides additional stimuli for cell proliferation and survival without any xenogenic components. However, higher concentrations of serum can inhibit cell growth.^4^ Moreover, its limited supply and lot-lot variability will ultimately impede progress in CAR-T cell-based therapies over time. The metabolic composition of serum varies in a species-specific manner. For example, uric acid, a metabolic end product that inhibits nucleotide biosynthesis, is 10-fold lower in human relative to murine or bovine serum.^5^ As uric acid impedes human cell proliferation, its omission from media formulations for cell therapy is advised.

Many paracrine, systemic, and metabolic factors with known roles in cell differentiation originate in erythrocytes, endothelial cells and platelets; cells commonly found in plasma. This led us to question whether extracts from cells found in transfusion grade whole blood or whole blood fractions can effectively support T cell differentiation. For instance, platelets contain a rich source of growth factors that support stem cell replenishment and differentiation in other cell types.^6^^,^^7^ In regenerative medicine, human platelet lysate (hPL), which is produced by freezing and thawing human platelets to release growth factors and trophogens in a lysate, is an effective growth factor supplement for several cell types including articular chrondrocytes, endothelial cells, dendritic cells, and osteoblasts.^3^ hPL enhanced corneal endothelial cell proliferation and survival *in vitro* relative to FBS.^8^ In clinical settings, platelet enriched plasma provides an important source of trophogens and growth factors facilitating stem-cell-mediated tissue regeneration and repair. Increasing evidence supports a role for platelet-derived growth factors (PDGFs) in mesenchymal stem cell renewal during *ex vivo* culture.^9^ PDGFs have also been implicated in the renewal and differentiation of multipotent stem cells participating in neurogenesis.^10^^,^^11^ Expressing PDGF in stem cells during *ex vivo* culture improved the corresponding potency following transplantation in a rat model of cardiac ischemia.^12^ It is well established that limiting CAR-T cell differentiation during the *ex vivo* expansion phase gives rise to progeny with increased therapeutic potential.^13^ Of note, anti-Erb2 CAR natural killer (NK)-92 cells have been successfully grown using hPL.^14^ Additionally, two very recent studies have shown that human T cells can be expanded in medium conditioned with hPL.^8^^,^^15^

Another component in blood that plays a role in supporting T cell proliferation are human red blood cells. Red blood cell conditioned media contains hemoglobin and peroxiredoxin II; regulatory factors permissive for T cell proliferation.^16^ In other studies, up to 46 cytokines and chemokines have been measured in red blood cells.^17^ We hypothesized that a serum free, concentrated growth factor extract, purified from human transfusion-grade blood fractions, will support *ex vivo* CAR-T cell transduction and preserve beneficial subsets responsible for long-lasting immunity against cancer.

We describe a novel system for transducing and stimulating CAR-T cells using a serum-free approach. We show that a concentrated growth factor extract, purified from human transfusion grade whole blood fractions, can effectively replace serum in CAR-T cell cultures. We found that CAR-T cell transduction is significantly enhanced in medium conditioned with a concentrated growth factor extract relative to HS. Anti-GD2 CAR-T cells expanded in medium supplemented with Phx demonstrate increased cytotoxicity *in vitro*. This contributes to an increased therapeutic potency in xenograft models of neuroblastoma. We provide evidence that CAR-T cells expanded in Phx also have superior engraftment and survival following transplantation *in vivo*. As *ex-vivo* culture forms the foundation for many adoptive immunotherapies, the development of customized serum-free formulations, with or without chemically defined derivatives that conserve cell bioactivity and function will lead to rapid advances in CAR-T therapies.

## Results

Several strategies have been developed to increase the effectiveness of CAR-T cells against cancer. Modifying CAR design, limiting differentiation, and streamlining the manufacturing process, have all increased the clinical impact of CAR-T cells. Optimizing the media formulation to support the expansion of CAR-T cells with increased potency is a simple and untested approach. In this study, we hypothesized that the unique composition of paracrine, systemic, and metabolic factors within a concentrated growth factor extract, which was purified from transfusion grade blood fractions, would enhance CAR-T cell potency.

**Figure 1:**
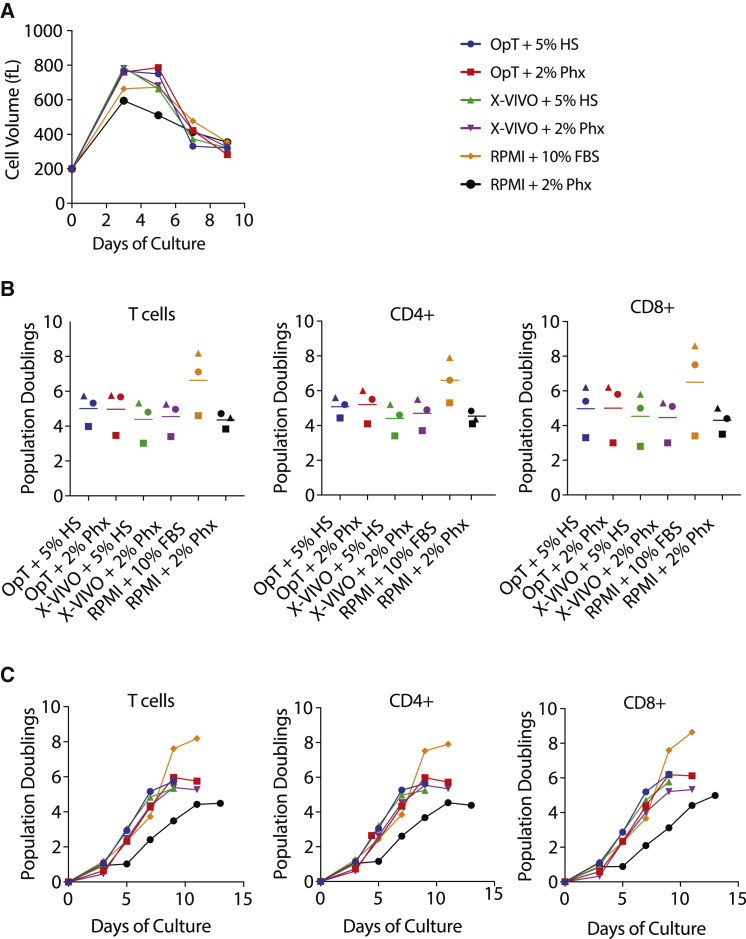
The Concentrated Blood Cell Extract, Physiologix, Supports Primary Human T Cell Expansion in Clinical and Research-Grade Medias (A) A mixed population of T cells were stimulated with anti-CD3/CD28 Dynabeads and expanded in medium conditioned with 5% human serum or 2% Physiologix. The mean cell volume was measured every other day beginning on day 3 until the number of cells in the culture ceased increasing and the mean cell volume is below 350 fL. Representative data from 3 independent experiments are shown. (B) T cells were stimulated as in (A). Cell enumeration was performed every other day beginning on day 3 until the number of cells in the culture ceased increasing and the mean cell volume is below 350 fL. The maximum number of population doublings is plotted with the horizontal bars representing the mean and each symbol representing a separate donor. Data were analyzed by a one-way ANOVA with Neuman-Keuls multiple comparison test. (C) T cells were stimulated as in (A). Cells were enumerated at the indicated time points. Data presented for bulk T cells, as well as CD4^+^ and CD8^+^ T cell subsets, are representative of 3 independent experiments with separate donors.

Activated T cells form large blasts in preparation for cell division. During this blast phase, T cells synthesize and accumulate macromolecular components for their daughter cells. To test the impact of Phx on T cell activation and proliferation, we activated T cells using anti-CD3/CD28 Dynabeads in media containing standard concentrations of 5% HS or 2% Phx. We chose a final concentration of 2% growth factor extract based on its previously described use in mesenchymal stem cells and CD4^+^ T cells.^18^ As seen in Figure 1A, the mean cell volume, a proxy of activation, was similar between T cells stimulated in media containing standard concentrations of 5% HS or 2% Phx. Cell size 5 days after activation was similar across all groups; measuring 705 ± 23 fL (OpT+HS), 719 ± 34 fL (OpT+Phx), 711 ± 26 fL (X-VIVO+HS), and 695 ± 19 fL(X-VIVO+Phx). There was a modest increase in blast size in cells cultured in RPMI + FBS relative to Phx (631 ± 21 fL versus 518 ± 4.9 fL). This blast phase is associated with an ensuing proliferative burst following T cell activation.

Following direct or surrogate antigen stimulation, activated T cells enter a logarithmic phase of expansion. During this proliferative phase, T cells are propagated to increase their quantity prior to adoptive cell transfer. We rigorously tested the effect of Phx on T cell proliferation and survival. We found that primary human T cells exhibit similar proliferative capacity when clinical grade media OpTmizer or X-Vivo-15 media is formulated with either 2% Phx or 5% HS (Figure 1B). In OpTmizer medium supplemented with 2% Phx, the average number of population doublings per cell, over 9–11 days, is 5.8 ± 0.8. T cells expanded in OpTmizer + 5% HS exhibited an average population doublings of 5.7 ± 0.5. Similar findings were obtained in X-Vivo-15 media supplemented with 2% Phx or 5% HS (5.3 ± 0.6 and 5.3 ± 0.7, respectively). Identical trends were observed in CD4^+^ and CD8^+^ T cell subsets. T cell proliferation is modestly diminished when 2% Phx (maximal population doublings of 4.7 ± 0.3) is substituted for 10% FBS (maximal population doublings 8.2 ± 1.1) in RPMI medium. While the duration of log-phase growth is unaffected, overall proliferation is decreased (Figure 1C). This effect is mitigated by raising the total protein content of the medium with HS albumin (data not shown).

**Figure 2:**
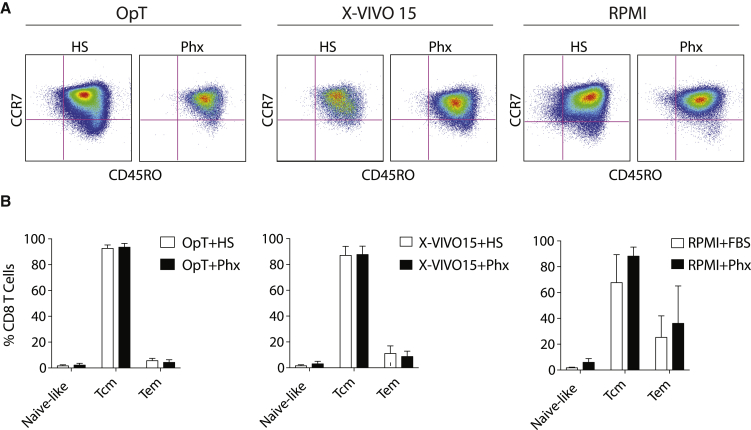
Differentiation Is Similar in T Cells Expanded in Medium Conditioned with Phx versus Human Serum Anti-CD3/CD28 stimulated T cells were expanded over several days in various media conditioned with either 5% human serum or 2% Physiologix. (A) As cells exit their proliferative phase, surface expression of CCR7 and CD45RO were measured by flow cytometry. Representative plots (n = 3) are shown. Cells were pre-gated for live, CD8^+^ T cells. (B) Relative proportion of naive-like (CCR7^+^; CD45RO^−^), central memory T cells (Tcm) (CCR7^+^; CD45RO^+^), and effector memory T cells (Tem) (CCR7^−^; CD45RO) subsets (after gating on live, CD8^+^ T cells). Data are mean ± SEM from 3 independent experiments.

As the CD4/CD8 ratio is an important aspect of T cell phenotype, we evaluated the *ex-vivo* expansion of a mixed population of CD4^+^ and CD8^+^ T peripheral blood T cells. To examine whether proliferation was differentially modulated within the CD4 or CD8 compartment, we evaluated the surface phenotype of cells prior to expansion, during expansion, and at the end of the log phase of expansion using bead-based flow cytometry. Table S1 shows that the ratio of CD4 to CD8 T cells was comparable between stimulatory conditions. Cell culture models are also informative about differentiation state. We show that T cells expanded in medium supplemented with either FBS, HS, or Phx possess similar levels of central memory and effector memory surface markers at the end of the *ex vivo* culture process (Figures 2A–2D).

**Figure 3:**
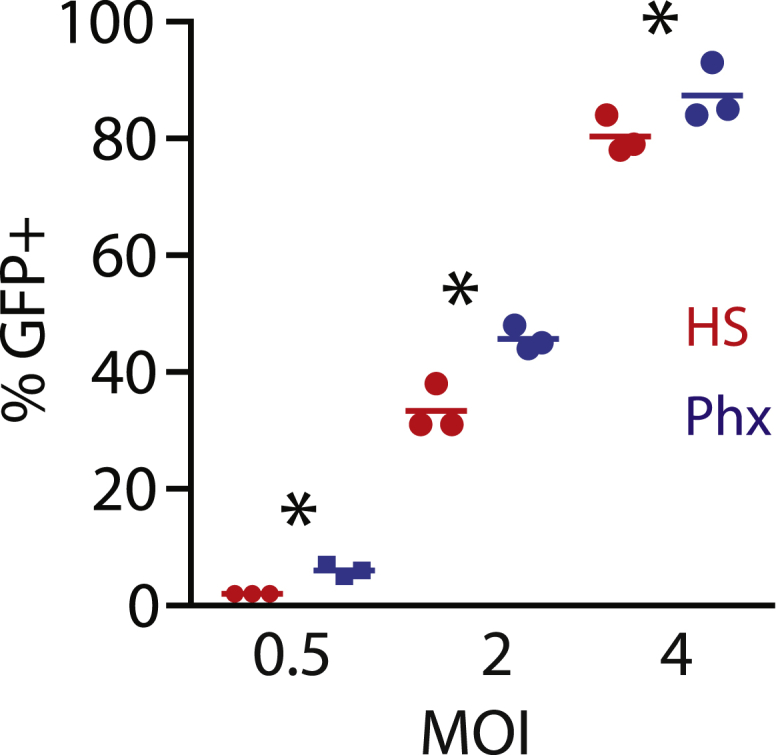
Phx Enhances Lentiviral-Mediated Gene Expression A mixed population of T cells were activated with anti-CD3/CD28 Dynabeads and cultured in OpTmizer medium conditioned with either 5% human serum or 2% Physiologix. Following overnight stimulation, previously titered GFP lentiviral supernatant was added at various dilutions. After 3 days, lentiviral infection efficiencies were measured by flow cytometry. The mean number of GFP^+^ cells ± SEM from 3 independent experiments with separate donors is shown. Data were analyzed by one-way ANOVA (p < 0.05). All groups were compared using a Newman-Keuls multiple comparison test with the difference between the HS and Phx groups statistically significant as indicated.

In CAR-based therapies, T cells are genetically modified with receptors that control antigen specificity. Lentiviral vectors are commonly used as vehicles to deliver CAR genes to activated T cells. Mechanistically, lentiviruses enter the cell and traverse the cytoplasm by endocytosis. Extrinsic growth factors and cytokines, which are abundantly different in Phx versus HS (Figure S1), can promote vesicle traffic king, mainly thorough the recruitment and activation of the Rab family of small GTPases.^19^ Access to metabolites can then support the reverse transcription of lentiviral genetic material into cDNA. To evaluate the effect of Phx on lentiviral gene transfer, we infected activated T cells with a lentiviral vector encoding EGFP and measured transduction after 72 h. At an MOI of 0.5, the number of GFP-positive cells was significantly increased (2% ± 0% versus 6% ± 0.6%, p < 0.05) when OpTmizer medium was conditioned with Phx rather than HS (Figure 3). Similar results were found at higher MOIs (Figure 3). These findings provide evidence that media supplemented with Phx enhance the transduction efficiency.

**Figure 4:**
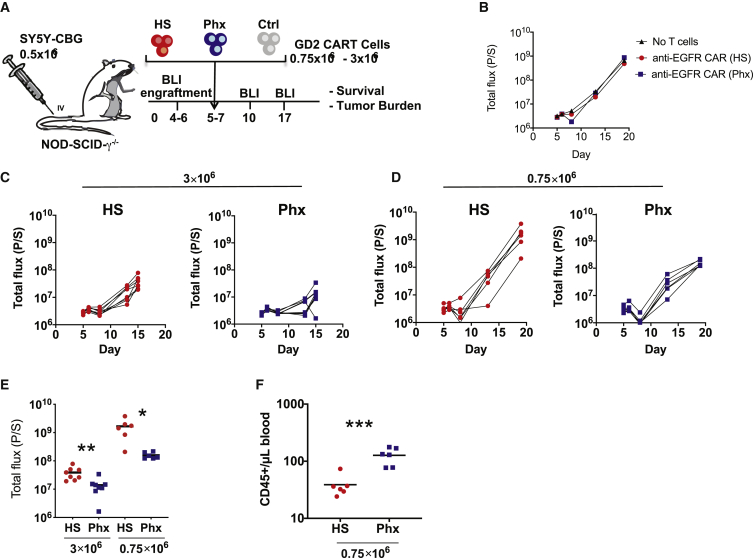
Investigating the Anti-Tumor Function of Anti-GD2 CAR-T Cells that Were Expanded in Medium Conditioned with Phx *In Vivo* (A) Schematic of the xenograft model and anti-GD2 CAR-T cell treatment (derived from healthy donors) in NSGs i.v. injected with 0.5 × 10^6^ SY5Y cells. Activated T cells were infected with anti-GD2 CAR lentivirus and expanded in OpTmizer medium conditioned with H.S. or Phx over 9 days. A high (3 × 10^6^) or a low (0.75 × 10^6^) dose of CAR^+^ T cells were selected to examine the influence of Phx versus HS on CAR-T cell potency. These cells were i.v. injected into NSG mice 5 days after SY5Y injection (n = 6–8 per group). (B–H) Serial quantification of disease burden by luminescence imaging. (B) Tumor size in mice that received no T cells, or T cells expressing an irrelevant CAR engineered to recognize human EGFR. Symbols represent a single mouse each. (C) The effectiveness of 3 × 10^6^ anti-GD2 CAR-T cells expanded in Phx versus anti-GD2 CAR-T cells expanded in HS. (D) The effectiveness of 0.75 × 10^6^ CAR-T cells expanded in Phx versus anti-GD2 CAR-T cells expanded in HS. (E) Quantification of tumor burden by bioluminescence imaging on days 10 (3 × 10^6^) and 14 (0.75 × 10^6^) in mice treated with anti-GD2 CAR-T cells. Values represent mean ± SEM for each group. (F) Blood was collected by retroorbital bleeding 14 days after tumor injection, and absolute peripheral blood CD45^+^ T cells were enumerated by a TruCount assay.

**Figure 5:**
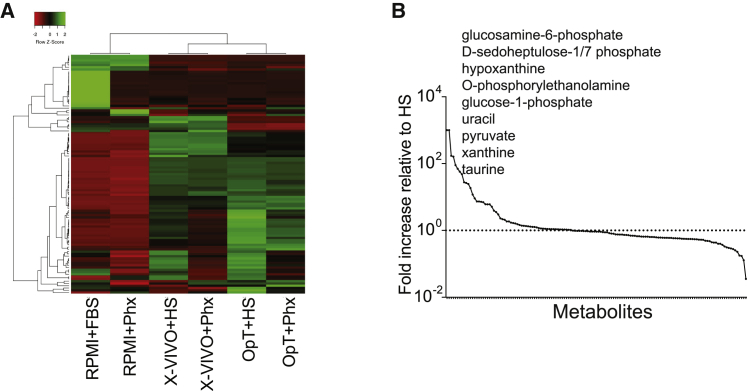
Metabolomic Assessment of OpTmizer Medium Conditioned with Human Serum Versus Phx (A) Heatmap illustrating the hierarchical relationship between metabolites present in various media conditioned with either human serum or Phx. In this grid, each row represents a unique metabolite and each column corresponds to a unique media formulation. (B) Metabolite abundance in Phx normalized to HS. The levels of metabolites present in both media were normalized to the level of HS. Metabolites were then rearranged in the x axis by the highest to lowest difference between metabolite levels derived from both supplementation methods. The dotted line represents the levels present in HS, while the solid line represents Phx.

In order to evaluate the functional consequences of Phx-based CAR-T cell manufacturing, we next evaluated the anti-tumor function of CAR-T cells expanded in Phx in a xenograft model of neuroblastoma. A schematic of the xenograft model is illustrated in Figure 4A. Given our earlier work in this area, we chose the E101K, anti-GD2 CAR engineered with a 4-1BB costimulatory domain for this study.^20^ Anti-GD-2 CAR-T cells were expanded in medium conditioned with 2% Phx versus 5% HS for 9 days (Figure S2). Consistent with our earlier findings using EGFP lentivirus, GD2 CAR expression levels were increased from 60% in HS to 73%. Immunodeficient mice were transplanted with Click Beetle Green Luciferase-expressing SY5Y cells. Tumor cell engraftment was confirmed 5 days later by bioluminescence. As seen in Figure 5A, initial tumor burden was identical across all experimental groups. We compared the potency of CAR-T cells expanded in HS versus Phx by infusing either 3 × 10^6^ or 0.75 × 10^6^ CAR-T cells intravenously (i.v.) and measuring the corresponding tumor size by bioluminescence at regular intervals. As expected, untreated SY5Y xenografts grew exponentially over time and control T cells expressing an irrelevant CAR had minimal impact on tumor cell growth (Figure 4B). A high dose (3 × 10^6^ cells) of CAR-T cells expanded *ex vivo* in HS significantly reduced tumor burden, as expected. However, GD-2-specific CAR-T cells, expanded *ex vivo* in Phx, demonstrated significantly superior tumor control (Figure 4C) To further evaluate potency in this model, we conducted a “stress test” by reducing the infusion dose of CAR-T cells to 0.75 × 10^6^ cells. Use of this “stress test” model is increasingly recognized as an important experimental parameter to provide insight into CAR-T cell potency.^13^^,^^21^ A lower dose of HS CAR-Ts led to incomplete tumor control compared with Phx CAR-Ts (Figure 4D). These findings provide evidence that Phx imbues CAR-Ts with increased anti-tumor potency in xenograft models (Figure 4E).

At the end of the experiment, we collected peripheral blood samples from mice treated with HS CARTS and Phx CAR-Ts. Circulating numbers of T cells were almost undetectable in the HS condition (Figure 4F). These findings are in agreement with prior research using this model.^20^ Circulating Phx-treated T cell numbers were significantly increased; an effect likely explained by superior replicative capacity and survival *in vivo*. The increased persistence of CAR-T cells following adoptive transfer contributes to enhanced tumor control, underscoring the functional nature of Phx relative to HS. Enhanced effector function may also contribute to the observed increase in tumor regression following Phx conditioning. To determine the role of Phx in CAR-T cell effector function, we compared the killing ability of Phx versus HS CARTs in a luciferase-based *in vitro* cytotoxicity assay. Following co-culture with GD-2 expressing SY5Y target cells, Phx CAR-induced cytolytic activity is significantly enhanced relative to HS CAR-T cells across a wide range of effector:target (E:T) ratios (Figure S3).

Given the renewed interest in understanding how metabolites influence T cell differentiation and effector function,^22^ we compared the metabolite content of Phx versus HS. Using an untargeted metabolomics approach, we screened Phx versus HS to reveal factors unique to Phx that could contribute to differences in gene transduction and overall potency. We found that several monosaccharide derivatives including glucosamine-6-phosphate, D-sedoheptulose-1/7 biphosphate, and glucose-1 phosphate were increased by several orders of magnitude in Phx relative to HS (Figures 5A and 5B). Phospholipid and nucleotide derivatives were also increased in Phx relative to HS including O-Phosphoethanolamine and hypoxanthine, xanthine, and uracil, respectively. Factors with well-established roles in central metabolic pathways including pyruvate and taurine were more abundant in Phx.

**Figure 6:**
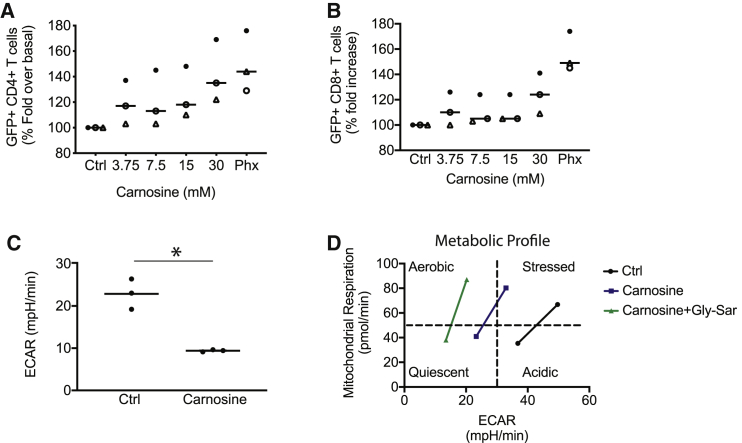
Carnosine Enhances GFP^+^ Lentivirus Expression and Improves the Metabolic Properties of T Cells (A and B) Freshly isolated T cells were activated in cell culture medium containing human serum and supplemented with varying levels of carnosine. As a positive control, T cells were activated in cell culture medium containing 2% Phx. After 24 h, T cells were infected with GFP lentivirus. The proportion of live, GFP^+^, T cells in the CD4^+^ (A) and CD8^+^ (B) compartment are shown. T cell subsets were enumerated using bead-based flow cytometry, as described in the Materials and Methods. Values are expressed as a percentage of OpT+HS. Mean ± SEM from 3 independent experiments with separate donors are shown. (C) T cells were activated with Dynabeads. After overnight stimulation, the cells were switched to a bicarbonate-free XF assay medium containing 30 mM carnosine. Metabolic parameters were measured by extracellular flux assay. Steady state extracellular acidification rates (ECARs) are shown. Values are mean ± SEM from 3 independent experiments with separate donors. ∗p < 0.01 for control versus carnosine as analyzed by an unpaired Student t test. (D) T cells were activated with Dynabeads for 1–3 days. The cells were switched to a bicarbonate-free XF assay medium containing 10 mM carnosine and 30 mM glycylsarcosine (Gly-Sar). Representative data from two independent experiments with separate donors are shown.

Our metabolomics screen identified a dipeptide containing β-alanine and L-histidine, commonly known as carnosine, that was 166% higher in Phx relative to HS (Figures 5A and 5B). To determine whether carnosine enhances lentiviral gene expression in activated T cells, we supplemented HS-based culture medium with various concentrations of carnosine (3.75–30 mM). These concentrations are similar in magnitude to other studies.^23^^,^^24^ GFP lentiviral supernatant was added to carnosine-treated T cells at an MOI of 0.5. We observed a dose-dependent increase in gene transduction up to 30 mM; at this concentration, carnosine effectively increased the number of T cells expressing GFP from 31% to 43% (p = 0.05; Figure 6A). Interestingly, this effect was more in the CD4^+^ T cell compartment. Our data introduce this histidine dipeptide as an important factor that enhances lentiviral-mediated CAR transduction in activated T cells.

Strategies to limit glycolytic metabolism during *ex vivo* culture have led to T cells with enhanced anti-tumor function.^25^, ^26^, ^27^ An underlying premise of these studies is that glycolysis, through poorly understood mechanisms, gives rise to effector differentiated T cells; a subset with poor engraftment and persistence following adoptive transfer. Glycolysis culminates in lactic acid (H^+^La^−^), which dissociates into the oxidizable fuel source, lactate, and a corresponding proton. Both La^−^ and H^+^ are exported extracellularly. The detrimental effect of glycolysis-induced acidification, which has intracellular and extracellular consequences, on T cell function and quality is unknown.

To test the ability of carnosine to neutralize extracellular acidification, we performed an extracellular flux (Seahorse) assay. After overnight stimulation with Dynabeads, T cells were switched to a Seahorse assay medium containing HEPES, and supplemented with 30 mM carnosine. The pH of the medium was adjusted to pH 7.4 prior to use. As seen in Figure 6C, carnosine effectively buffers extracellular proton accumulation; significantly decreasing the steady state extracellular acidification from 23 to 9 mpH/min (p < 0.05). Carnosine also shifts the metabolic profile of activated T cells from a glycolytic to an oxidative state (Figure 6D). An oxidative metabolic profile is superior to a glycolytic metabolic state during cell culture, as it culminates in T cell progeny with superior anti-tumor function.^25^, ^26^, ^27^ To distinguish between an intracellular versus extracellular benefit of carnosine, we included Gly-Sar to competitively inhibit carnosine uptake via the cell surface transporter PEPT1. Gly-Sar accentuates the ability of carnosine to limit extracellular acidification and enhances the impact of carnosine on the overall metabolic phenotype (Figure 6D).

Taken together, these findings highlight the therapeutic potential of Phx as a preferred alternative to HS; yielding enhanced CAR gene delivery and potency in xenograft models of cancer. Our findings also highlight the therapeutic merit of identifying and including biogenic amines such as carnosine, which was modestly enriched in Phx relative to HS, to improve the overall quality of the cell culture medium for CAR-T cell therapies.

## Discussion

We show that CAR-T cells grown in Phx demonstrate increased potency relative to HS. Anti-GD2 CAR-T cells expanded in Phx were functionally superior to CAR-T cells expanded in HS; an effect accentuated when limited numbers of infused CAR-Ts were compared. The increased accumulation of Phx-treated T cells in peripheral blood, coinciding with tumor clearance, is a likely consequence of enhanced replicative capacity, cytolytic activity, and survival. It is unlikely that differences in CAR-T cell activation contribute to their superior therapeutic impact as the overall antigen density was identical across all groups, prior to CAR-T infusion.

Our data encourages further optimization of media formulations used in adoptive immunotherapies. We show that Phx enhances the potency of CAR-T cells redirected against the solid tumor GD-2 antigen. Expanding CAR-T cells in medium conditioned with growth factor extracts enhances their persistence and effector function following transplantation. As most cells are excluded from serum used in cell culture, our findings raise important questions in media design—are current *ex vivo* media formulations suboptimal for CAR-T cell function? Are CAR-T cells hindered by the conditions in which we expand them *ex vivo*? Our data may accelerate future progress by including systemic factors permissive for enhanced engraftment following re-infusion.

We tested Phx as a serum-free alternative across a broad range of media. We showed that Phx is a suitable conditioning agent for use in research grade RPMI medium, as well as clinical grade X-Vivo15 and OpTmizer media. Media supplemented with Phx support the *ex vivo* expansion of T cells isolated from healthy volunteers. A modest reduction in T cell proliferative capacity was observed in RPMI supplemented with 2% Phx versus 10% FBS. At these concentrations, stoichiometric imbalances in bioactive peptides, growth factors, and lipids may underlie this effect.

Activated T cells are genetically modified with CARs that confer antigen specificity, direct signal transduction, promote effector function, and reprogram metabolism. CAR gene delivery has been optimized through the use of HIV-based lentiviral plasmids but remains costly in the clinical domain. Lentiviral-mediated gene delivery is significantly enhanced 2- to 3-fold in cells activated in medium conditioned with Phx. Our findings implicate a role for carnosine, which is modestly elevated in Phx relative to HS, in this process. Other factors which are abundant in Phx, but remain untested, may also support this process.

The effector:target ratio reflects the dynamic equilibrium between the number of functionally competent T cells and their corresponding targets. A number of T cell intrinsic factors including replicative capacity, differentiation, and survival combine to shift the equilibrium in favor of T cell cytolytic activity over tumor cell proliferation. At an infusion dose of 0.75e6, only CAR-T expanded in Phx surpassed the critical set point necessary to control tumor burden. These findings show that media formulation is an important parameter influencing CAR-T cell efficacy.

There is renewed interest in understanding how metabolism regulates CAR-T cell efficacy, with important implications for improving the quality of T cells used in adoptive immunotherapy. Surprisingly, the metabolite content of HS, an essential component of clinical grade media, is largely uncharacterized. We provide novel evidence that carnosine, which is modestly increased in Phx relative to HS, effectively neutralizes the extracellular proton load in activated T cells. Our findings are impactful as CAR design can exacerbate extracellular acidification in culture; CARs designed with CD28 costimulatory domains accentuate glycolytic metabolism and its corresponding byproduct lactic acid.^28^ Previously, carnosine has been shown to limit intracellular acidification in skeletal muscle cells undergoing high rates of glycolysis,^29^ and our findings extend its roles to T cells.

Plasma carnosine levels are low due to the activity and abundance of carnosinase (CN) enzymes. We show that transiently raising carnosine levels in HS-based culture medium improves lentiviral gene expression. The metabolic “fitness” of carnosine-treated T cells also increases; exemplified by a shift from a stressed/acidic state toward an oxidative/energetic state. Given the intrinsic ability of carnosine to sequester protons, inclusion of carnosine in medium formulations may enhance the quality of T cells as they undergo Warburg metabolism during *ex vivo* activation.

Future studies will shed more light on how the metabolome of blood cell growth factor extracts, rather than serum, support enhanced CAR-T cell gene delivery and potency. The potential of improved transduction and *in vivo* performance, even when using lower amounts in final media formulations, is an attractive benefit of these products. In one analysis on the direct bioprocessing costs for CAR T cells, activation and transduction were the majority of the cost. Depending on the bioprocessing vessel these two attributes could be as much as 85% of the overall direct cost of goods sold (COGS). This same study confirmed that variability in proliferation, contrary to other studies, had the least effect on overall COGS.

In conclusion, our work provides evidence that CAR-T cell potency can be further enhanced by extrinsic factors present in the media during the manufacturing process. These findings shed light on mechanisms that regulate gene transfer, metabolic fitness, effector function, and persistence; processes that are essential for CAR-T *in vivo* behavior and may lead to stepwise improvements in *ex vivo* T cell production for therapeutic purposes.

## Materials and Methods

### Cell Culture

Primary human leukocytes (PBLs) from healthy male and female volunteers, averaging 28 ± 1.3 years of age, were collected at the University of Pennsylvania’s Apheresis Unit. Informed consent was obtained from all participants before collection. All experimental procedures and methods were approved by the University of Pennsylvania Institutional Review Board. T lymphocytes were purified at the University’s Human Immunology Core by negative selection using the RosetteSep T cell enrichment cocktail (Stemcell). After isolation, T cells were cultured in various growth media described in detail in Table 1. OpTmizer and RPMI 1640 were purchased from GIBCO. X-VIVO15 was obtained from Lonza. These media were conditioned with either 5% human AB serum (Gemini Bioproducts), 10% FBS (Hyclone), or 2% Physiologix (Nucleus Biologics). All media were supplemented with 2 mM L-glutamax, 100 U/mL penicillin G, and 100 μg/mL streptomycin. All cells were grown in standard conditions using a humidified incubator maintained at 5% CO_2_ and 37°C. To activate T cells, we used 4.5 μm Dynabeads bearing immobilized anti-human CD3 and anti-human CD28 (Life Technologies) at a ratio of 3 beads to 1 T cell. Lymphocytes were maintained in culture at a concentration of 0.8–1.0 × 10^6^ cells/mL through regular counting by flow cytometry using CountBright beads (BD Biosciences), a viability marker (Viaprobe) and mAbs to either human CD4 or CD8 as described in O’Connor et al.^30^ T cell size was measured on a Multisizer ΙΙΙ particle counter (Beckman-Coulter). After 9 days in culture, T cells were magnetically debeaded, washed in PBS, and resuspended in standard freezing medium comprising 5% DMSO and 95% FBS prior to cryopreservation. The SY5Y-click beetle green luciferase-expressing tumor cells were generated as described previously.^20^

**Table 1:** Media Composition

	OpTmizer+ 5% HS	OpTmizer+ 2% Phx	X-VIVO15+ 5%HS	X-VIVO15+ 2%Phx	RPMI 1640+ 10%FBS	RPMI 1640+ 2%Phx
Base medium	✓	✓	✓	✓	✓	✓
HS	5%		5%			
OpT supplement	2.6%	2.6%				
FBS					10%	
Phx		2%		2%		2%
Glutamax	2 mM	2 mM	2 mM	2 mM	2 mM	2 mM
P/S	1%	1%	1%	1%	1%	1%
HEPES					1%	1%

### Antibodies and Flow Cytometry

Activated T cells were expanded for 9–11 days and subsequently stained with a panel of monoclonal antibodies to assess differentiation status. The following pre-titrated antibodies were used: anti-CCR7–FITC (clone 150503; BD PharMingen); anti-CD45RO-PE (clone UCHL1), anti-CD8-H7APC (clone SK1; BD Biosciences); anti-CD4-BV605 (clone OKT4). 1 × 10^6^ cells were immunostained as follows: cells were washed with phosphate-buffered saline (PBS) and stained for viability using LIVE/DEAD Fixable aqua (Molecular Probes) for 15 min, washed once, and resuspended in fluorescence activated cell sorting (FACS) buffer consisting of PBS, 1% BSA, and 5 mM EDTA. Cells were then incubated with the above indicated antibodies for 30 min at room temperature (RT). Samples were then washed three times with FACS buffer and fixed in 1% paraformaldehyde. Positively stained cells were differentiated from background using fluorescence-minus-one (FMO) controls. Flow cytometry was performed on BD LSR Fortessa. Analysis was performed using Flowjo software (Tree Star version 10.1).

### Lentiviral Production

The lentiviral vector pELNS-GFP encodes EGFP under the transcriptional control of EF-1α. Lentiviral supernatants were generated by transient transfection of 293-T cells with pELNS-GFP. 293-T cells were initially seeded in T150 flasks and grown to 80% confluence in 25 mL of culture medium (RPMI 1640). 90 μL Lipofectamine 2000 DNA transfection reagent was combined with 7 μg pCL-VSVG, 18 μg pRSV-REV, and 18 μg of pGAG-POL (Nature Technology) as well as 15 μg of pELNS-GFP. This mixture was incubated at RT for 15 min. DNA-Lipofectamine complexes were then added to the 293-T cells. After 24 h, infectious supernatants were sterile filtered through a 0.45-μm syringe tip cellulose acetate filter and collected in a 50 mL conical tube. To pellet the lentivirus, we spun the supernatant in a Thermo Fisher Scientific Centrifuge (LYNX 4000) at 18,000 RCF, overnight, at 4°C. The supernatant was removed and the lentiviral pellet was resuspended in 1.6 mL of culture medium, aliquoted, and stored at −80°C. Generation of the GD2-E101K CAR lentiviral plasmid was previously described in Richman et al.^20^ The CAR contains the 14G2a single chain variable fragment (scFv), as well as an EF1α promoter, CD8 hinge, 4-1BB costimulatory domain, and CD3ζ signaling domain. The anti-EGFR CAR, with 3C10 scFv, was previously described in Johnson et al.^31^

### Lentiviral Infection

Prior to activation, freshly isolated human T cells were resuspended in the following media: OpTmizer medium supplemented with 5% HS, OpTmizer + 2% Physiologix, X-VIVO 15 + 5% HS, X-VIVO 15 + 2% Physiologix, RPMI 1640 + 10% FBS or RPMI1640 + 2% Physiologix. T cells were activated with Dynabeads as described above. 24 h after activation, T cells were seeded at 100,000 cells/well at a concentration of 1 × 10^6^ cells/mL in a 96-well culture dish. Serial dilutions of lentiviral supernatant over a range of 1:3, 1:9, 1:27, 1:81, 1:243, and 1:729 were performed. Infected T cells were grown for 72 h to ensure optimal gene expression before comparing transduction efficiencies. The percentage of GFP^+^ cells was determined by flow cytometry and the corresponding titer was calculated as the number of transforming units/mL.

### CAR Expression

2 × 10^6^ cells T cells were activated with Dynabeads and cultured in OpTmizer medium conditioned with either 5% HS or 2% Phx at a concentration of 1 × 10^6^ cells/mL. Following overnight stimulation, 200 μL anti-GD-2 lentiviral supernatant was added to the cultures. The lentiviral titer was previously calculated as 22.68 × 10^6^ transforming units/mL using RPMI 1640 medium containing 10% FBS. Infected cells were expanded over 5 days and harvested for CAR immunostaining. Briefly, 1 × 10^6^ cells were resuspended in FACS buffer and immunostained with 10 μL of a pre-titrated biotin-conjugated goat-anti-mouse immunoglobulin G (IgG) F(ab’)2 fragment (Jackson ImmunoResearch) for 20 min at RT. After a series of washes, the cells were immunostained with streptavidin-PE (Biolegend) for 20 min at RT. A final series of washes were performed and the cells were fixed in 1% paraformaldehyde diluted in PBS. Nontransduced cells, as well as cells stained with streptavidin-PE alone, were included as staining controls. The percentage of CAR^+^ cells was determined by flow cytometry. In parallel, 1 × 10^6^ T cells were activated with Dynabeads. After overnight stimulation, 100 μL of anti-EGFR lentiviral supernatant was added to the cultures. After 5 days, CAR expression was measured as described above after immunostaining with a biotin-conjugated goat-anti-human IgG F(ab’)2 fragment (Jackson ImmunoResearch) and streptavidin-phycoerythrin (SA-PE).

### *In Vitro* Cytotoxicity Assay

The ability of GD2-specific CAR-T cells to kill tumor targets was tested in a luciferase-based cytotoxicity assay. SY5Y cells engineered to stably express click beetle green luciferase were co-cultured with CART-GD2 cells or donor-matched untransduced (UTD) T cells at the indicated CAR^+^ T cells: target cell (E:T) ratios. GD-2-specific CAR T cells were expanded in either OpTmizer medium supplemented with 5% HS or OpTmizer supplemented with 2% Physiologix as described above. All T cells were expanded for 9–11 days in their respective media until restdown (cell size: 340–400 fL). Prior to the cytotoxicity assay, all T cells were washed in PBS and resuspended in serum free medium for 30 min. This medium was then replaced with standard killing assay medium (RPMI 1640+10% FBS) for the duration of the assay. 6 replicates per condition were seeded in 96-well round-bottom plates in a total volume of 200 μL. CAR induced cytolysis, as measured by a decrease in the luciferase signal generated by live target cells, was assessed at 20 h. Briefly, Luciferin (Promega) was added at a final concentration of 150 μg/mL per well. Luminescence was measured after a 10 min incubation using the EnVision (PerkinElmer) plate reader and luciferase activity was expressed as relative luminescent units (RLUs). Note that target cells incubated in medium alone or treated with 1% SDS were used to calculate spontaneous cell death (RLU_spon_) or maximal cell death (RLU_max_), respectively. The percent-specific lysis was calculated using the formula: % specific lysis = 100 × ([RLU_spon_– RLU_test_]/[RLU_spon_ – RLU_max_]). All data are presented as mean ± SEM.

### *In Vivo* Xenograft Studies

A xenograft model was used in this study as previously reported.^20^ Briefly, 6- to 10-week-old non-obese diabetic severe combined immunodeficiency (NOD-SCID) γ_c_^–/–^ (NSG) mice, which lack an adaptive immune system, were obtained from Jackson Laboratories (Bar Harbor, ME, USA) or bred in-house under a protocol approved by the Institutional Animal Care and Use Committees of the University of Pennsylvania. Animals were assigned in all experiments to treatment/control groups using a randomized approach. Animals were injected i.v. via tail vein with 0.5 × 10^6^ SY5Y-CBG tumor cells (ATCC) in 0.1 mL sterile PBS. On day 5, tumor engraftment was confirmed. Anti-GD2 CAR-T cells or anti-EGFR CAR-T cells and non-transduced UTD human T cells were injected i.v. at the indicated dose in 100 μL of sterile PBS, 5 days after injection of SY5Y-CBG tumor cells. Anesthetized mice were imaged using a Xenogen IVIS Spectrum system (Caliper Life Science) twice a week. Mice were given an intraperitoneal injection of D-luciferin (150 mg/kg; Caliper Life Sciences). Total flux was quantified using Living Image 4.4 (PerkinElmer) by drawing rectangles of identical area around mice, reaching from head to 50% of the tail length. Peripheral blood was obtained by retro-orbital bleeding in an EDTA coated tube, and blood was examined fresh for evidence of T cell engraftment by flow cytometry using BD Trucount (BD Biosciences). Animals were euthanized at the end of the experiment before showing signs of toxicity and before reaching signals higher 1 × 10^11^ p/s total flux per mouse (in accordance with our Institutional Animal Care and Use Committee [IACUC] protocols).

### Liquid Chromatography-Mass Spectrometry (LC-MS) Analysis

Metabolome extraction was performed by adding 30 volumes of 80:20 methanol/water to 3 μL of sample. After centrifugation at max speed (20 min, 4°C), clean supernatants were transferred to LC vials and analyzed using a quadrupole-orbitrap mass spectrometer (Q Exactive, Thermo Fisher Scientific, San Jose, CA, USA) coupled to hydrophilic interaction chromatography via electrospray ionization. LC separation was on a XBridge BEH Amide column (2.1 mm × 150 mm, 2.5 μm particle size; Waters, Milford, MA, USA) using a gradient of solvent A (20 mM ammonium acetate, 20 mM ammonium hydroxide in 95:5 water: acetonitrile, pH 9.45) and solvent B (acetonitrile). Flow rate was 150 μL/min, column temperature was 25°C, autosampler temperature was 5°C, and injection volume was 10 μL. The LC gradient was: 0 min, 90% B; 2 min, 85% B; 3 min, 75% B; 7 min, 75% B; 8 min, 70% B; 9 min, 70% B; 10 min, 50% B; 12 min, 50% B; 13 min, 25% B; 14 min, 25% B; 16 min, 0% B; 21 min, 0% B; 22 min, 90% B; 25 min, 90% B. Autosampler temperature was 5°C, and injection volume was 10 μL. The mass spectrometer was operated in negative ion mode to scan from m/z 70 to 1,000 at 1 Hz and a resolving power of 140,000. Data were analyzed using the MAVEN software.

### Cytokine and Growth Factor Assessment

HS versus Phx cytokine levels were analyzed with a Luminex bead array platform (Life Technologies) according to the manufacturer’s instructions. All samples were analyzed in triplicate and compared against multiple internal standards with a nine-point standard curve. Data were acquired on a FlexMAP-3D system (Luminex), and analysis was performed in XPonent 4.0 software (Luminex), as well as through five-parameter logistic regression.^32^

### Lentiviral Infection Efficiency following Carnosine Supplementation

Prior to activation, freshly isolated human T cells were resuspended in OpTmizer medium supplemented with either HS or Phx. OpT+HS was further supplemented with Varying doses of carnosine. The pH of all media was adjusted to 7.4 prior to use. T cells were then activated with Dynabeads as described above. T cells were seeded in duplicate at a concentration of 1 × 10^6^ cells per well in a 12-well culture dish. 24 h after activation, GFP lentiviral supernatant was added at an MOI of 0.5. Infected T cells were grown for 72 h to ensure optimal gene expression before comparing transduction efficiencies. The percentage of GFP^+^ T cells was determined by flow cytometry using mAbs to human CD4 and CD8, as well as a live-dead dye. All data were normalized to control (OpT+HS). Data from three independent donors in separate experiments are presented as mean ± SEM.

### Mitochondrial Respiratory Features

Mitochondrial function was assessed using an extracellular flux analyzer (Agilent/Seahorse Bioscience). Individual wells of an XF96 cell culture microplate were coated with Cell-Tak in accordance with the manufacturer’s instructions. The matrix was adsorped overnight at 37°C, aspirated, air-dried, and stored at 4°C until use. XF assay medium (non-buffered RPMI 1640) containing 10 mM glucose, 2 mM L-glutamine, and 5 mM HEPES was prepared immediately before the assay. 30 mM Carnosine (kindly provided by Nucleus Biologics) was added to this base medium and its pH was adjusted to 7.4. T cells were stimulated overnight with Dynabeads as described above. To assay mitochondrial function, we centrifuged activated T cells at 1,200 × *g* for 5 min and washed them in PBS. Cell pellets were re-suspended in XF assay medium and seeded at 0.2 × 10^6^ cell/well. During instrument calibration, the microplate was centrifuged at 1,000 × *g* for 3 min and switched to a CO_2_-free, 37°C incubator for 15 min only. Cellular oxygen consumption rates (OCRs) as well as extracellular acidification rates (ECARs) were measured under basal conditions and following treatment with 1.3 μM oligomycin and 1.5 μM fluoro-carbonyl cyanide phenylhydrazone (FCCP). ECAR levels (mean ± SEM) from three independent experiments with separate donors are shown. To compare the metabolic profile of T cells, we stimulated T cells with Dynabeads for 1–3 days. Activated T cells were resuspended in XF assay medium supplemented with 10 mM Carnosine, as well as 30 mM glycylsarcosine (Gly-Sar) purchased from Sigma. In all cases, the pH was adjusted to 7.4 prior to the assay. OCR and ECAR were measured as described above.

### Statistical Analyses

To determine statistical significance, we analyzed the mean data obtained from two groups using paired Student’s t tests. When comparing differences across multiple groups with a single independent variable, a one-way ANOVA with Newman-Keuls post-test comparisons were performed. Data from multiple groups with two independent variables were analyzed by two-way ANOVA using the Newman-Keuls post-test comparisons method. All analyses were performed using GraphPad Prism version 8.1.6e (GraphPad Software). For all tests, the significance of the difference between means was accepted as <0.05 level.

## Author Contributions

Conceptualization: S.G., F.J.M.-B., S.A.R., A.M.M., A.A., M.C.M., C.H.J., and R.S.O’C. Investigation: S.G., D.H., J.L., Y.T., J.C.G.C., F.J.M.-B., A.A., A.M.M, Y.L., R.S.O’C., and A.W. Writing – Original Draft: S.G. and R.S.O’C. Writing – Review & Editing: M.C.M. and J.D.R.

## Conflicts of Interest

S.G., M.C.M., and C.H.J. are inventors on several granted and pending patents related to CAR-T cells and their use for cancer therapy. R.S.O’C. is an inventor on a patent related to growing primary human T cells. S.A.R. is a co-inventor on a pending patent related to chimeric autoantigen receptor-engineered T cells. F.J.M.-B., A.M.M., and A.A. are employees of Nucleus Biologics. S.A.R., D.H., J.L., Y.T., J.C.G.C., Y.L., J.D.R., and A.W. declare no competing interests.

## Supplemental Information

Document S1. Figures S1–S3 and Table S1

Document S2. Article plus Supplemental Information
