# Supplementary material for: Enhancing Chimeric Antigen Receptor T Cell Anti-tumor Function through Advanced Media Design
Source: Mol Ther Methods Clin Dev. 2020 Jul 9;18:595–606. doi: 10.1016/j.omtm.2020.07.008 (PMC7397397; doi:10.1016/j.omtm.2020.07.008)
Supplement: Document S1. Figures S1–S3 and Table S1 [file mmc1.pdf]

OMTM, Volume *18*

Supplemental Information

Enhancing Chimeric Antigen Receptor T Cell Anti-tumor Function through Advanced Media Design

Saba Ghassemi, Francisco J. Martinez-Becerra, Alyssa M. Master, Sarah A. Richman, David Heo, John Leferovich, Yitao Tu, Juan Carlos García-Cañaveras, Asma Ayari, Yinan Lu, Ai Wang, Joshua D. Rabinowitz, Michael C. Milone, Carl H. June, and Roddy S. O'Connor

Supplemental

Figure S1

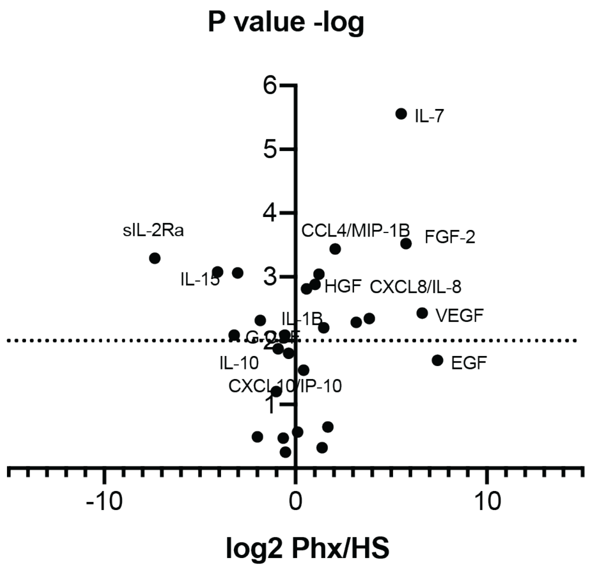

Fig S1. Cytokine and growth factor abundance in Phx relative to human serum

Cytokine levels in three independent lots of Phx were collected and compared to human serum using a 31-plex array. All samples were analyzed in triplicate and compared against multiple internal standards with a nine-point standard curve. Mean ± S.D.

Figure S2

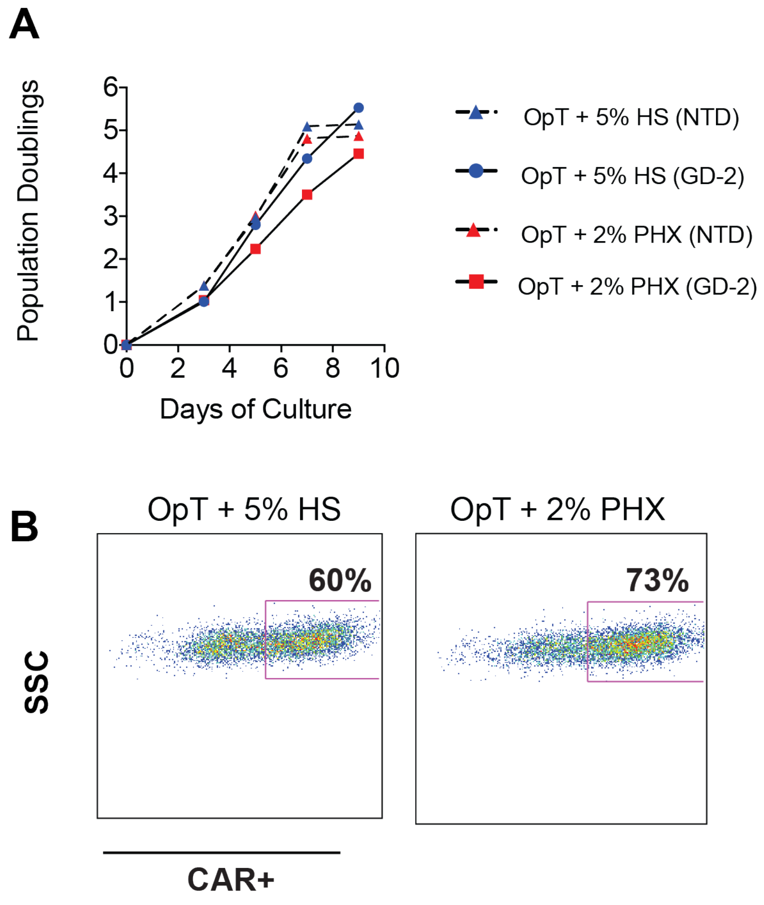

Fig S2. Anti-GD-2 CAR-T cell expansion

(A) A mixed population of T cells were stimulated with anti-CD3/CD28 Dynabeads and expanded in OpTmizer media conditioned with either 5% human serum or 2% Physiologix. Following overnight stimulation, activated T cells were lentivirally infected with either a GD-2-specific CAR transgene at an MOI of 4. In parallel, activated T cells were infected with an EGFR-specific CAR transgene at an identical MOI. Cells were enumerated at the indicated time points. (B) After 5 days, CAR expression levels were determined by immunostaining as described in *Materials and Methods*. The number of CAR+ cells are shown. Data are representative from several independent experiments.

Figure S3

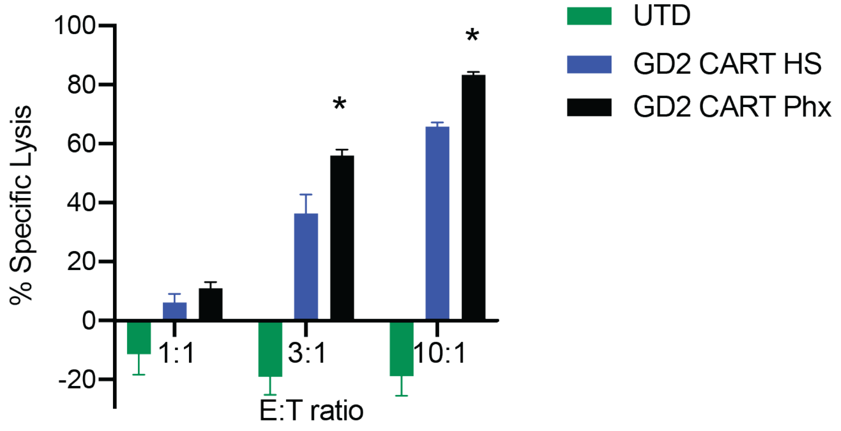

Fig S3. Investigating Anti-GD2 CAR-T cell cytotoxicity in vitro following prior expansion in Phx

The specific cytotoxicity of anti-GD2 CAR-T cells was measured by luciferase-based killing assay. CAR-T cells expanded in either OpT+HS or OpT+2%Phx were co-cultured with SY5Y target cells at the indicated E:T ratios for 20 hr in killing assay medium (RPMI 1640+10%FBS). The mean ± S.E.M. values of 6 replicates are shown. * $p<0.05$ for Phx vs HS at a 3:1 E:T ratio; ** $p<0.05$ for Phx vs HS at a 10:1 E:T ratio. Data were analyzed by a Two-way ANOVA using a Neuman-Keuls multiple comparison post-hoc test.

Table S1. T cell subset composition before and after expansion in various media

	**Start of the culture**		**OpT HS**	**OpT HS**	**OpT Phx**	**OpT Phx**	**X-VIVO HS**	**X-VIVO HS**	**X-VIVO Phx**	**X-VIVO Phx**	**RPMI HS**	**RPMI HS**	**RPMI Phx**	**RPMI Phx**
	CD4	CD8	CD4	CD8	CD4	CD8	CD4	CD8	CD4	CD8	CD4	CD8	CD4	CD8
**Donor 1**	47	38	69	31	63	36	58	43	54	44	58	40	59	41
**Donor 2**	69	25	65	35	68	32	65	34	74	26	62	38	64	36
**Donor 3**	N/A	N/A	47	52	45	55	42	57	46	53	35	65	60	39
